# Genotypes and Variants of BKPyV in Organ Donors after Brain Death

**DOI:** 10.3390/ijms23169173

**Published:** 2022-08-15

**Authors:** Jacek Furmaga, Marek Kowalczyk, Olga Furmaga-Rokou, Christos A. Rokos, Tomasz Zapolski, Leszek Krakowski, Andrzej Jakubczak, Sławomir Rudzki

**Affiliations:** 1 Department of General and Transplant Surgery and Nutritional Treatment, Medical University of Lublin, 20-954 Lublin, Poland; 2 Institute of Quality Assessment and Processing of Animal Products, University of Life Sciences in Lublin, 20-950 Lublin, Poland; 3 Department of Radiology, General Hospital of Thessaloniki George Papanicolaou, 56403 Thessaloniki, Greece; 4 Department of Otolaryngology, Head and Neck Surgery, AHEPA University Hospital, Aristotle University of Thessaloniki, Kiriakidi 1, 54636 Thessaloniki, Greece; 5 Department of Cardiology, Medical University of Lublin, 20-954 Lublin, Poland; 6 Department and Clinic of Animal Reproduction, Faculty of Veterinary Medicine, University of Life Sciences, Gleboka 30, 20-612 Lublin, Poland; 7 Institute of Biological Basis of Animal Production, Faculty of Animal Sciences and Bioeconomy, University of Life Sciences in Lublin, 20-950 Lublin, Poland

**Keywords:** BKPyV, kidney transplantation, viral load, genotyping, co-infection, cellular markers of inflammation, NLR

## Abstract

Kidney transplantation from a donor with latent BKPyV might be the cause of serious complications, such as BK virus-associated nephropathy. The aim of the study was to determine the prevalence of BKPyV infection in donors after brain death (DBDs), to analyse the molecular variation of BKPyV and to compare clinical and inflammation parameters of DBDs infected with various genotypes of BKPyV. BKPyV was investigated in blood and urine samples of 103 DBDs using PCR followed by sequencing and bioinformatic analysis, and the viral load was assessed by qPCR. Clinical parameters, including cellular markers of inflammation were assessed. The results confirm high prevalence of BKPyV (48%),and genotype IV (49%) over genotype I (43%) and the co-infection with genotypes I and IV in 8.2%. Viral load ranged from 10^2^ to 10^7^ copies/mL, with an average of 1.92 × 10^6^ copies/mL. No specific markers for BKPyV infection were detected among the parameters tested. Infection with genotype I may be associated with the adverse impact on thekidney function, while infection with genotype IV was associated with the anemia Not only the viral load but also the genotype of BKPyV may have an impact on the course of infection.

## 1. Introduction

Polyomaviruses (PyV) are widespread in the general population, but rarely induce overt clinical disease or pathology. This family of viruses coevolved with their hosts, as evidenced by their high prevalence and low morbidity [1]. PyV are latent viruses that can replicate in patients with acquired immunodeficiency. One of the representatives of the genus *Betapolyomavirus* of the family *Polyomaviridae* is the BK polyomavirus (BKPyV), which exhibits tropism for the renal tubules and cells of the transitional epithelium of the urinary tract. For this reason, kidney transplantation from a donor with latent BKPyV is associated with numerous serious complications. It can stimulate chronic inflammation, while active replication of the virus can lead to BK virus-associated nephropathy (BKPyVAN), which can result in loss of the graft [2]. Therefore, transplantation of BKPyV—infected kidneys should be carefully taken.

Concurrent studies are aiming to determine whether BKPyV replication is the result of reactivation of the pathogen in the recipient’s native kidneys or the effect of transmission of the virus from the allograft. The possibility of transmission of the virus along with the graft is confirmed by serological studies indicating a correlation between the donor’s seropositive status and replication in the recipient, as well as by molecular studies indicating high agreement of DNA sequences between BKPyV isolates from donors and recipients [3,4]. Thus, a growing body of research suggests that pre-transplant urinary BKPyV shedding in the donor is a risk for post-transplant infection, and therefore that donor-derived BKPyV transmission is an important mode of infection. For this reason, epidemiological data such as the genetic variation of the pathogen can help to understand the mechanisms associated not only with transmission of the virus, but also with the course of infection itself.

It is clear that due to its potentially serious complications, BKPyV infection in kidney donors, otherwise considered to be healthy, is of crucial importance for the function of the transplanted kidney and the health of the recipients. Urine cytology and quantitative polymerase chain reaction (qPCR) are primarily used for detecting BKPyV. However, the gold standard for diagnosing BKPyVAN is kidney biopsy, which may be associated with potential complications, sampling errors, and poor reproducibility [5].

Unlike in the case of living donors, it is usually not possible to perform detailed virological testing under the time pressure associated with transplantation from donors after brain death (DBDs) or with permanent irreversible cardiac arrest. Therefore, there is a need for simple and non-invasive screening in order to reduce the risk of organ transplant dysfunction associated with BKPyV infection [6].

There is increasing interest in better understanding of the indicators of inflammation, such as the neutrophil-to-lymphocyte ratio (NLR) [7,8], or platelet-to-lymphocyte ratio (PLR) which reflect the presence of systemic inflammation [9]. Both of them have been correlated with poor prognosis in a variety of conditions including end-stage kidney disease, heart disease or cancer [9,10,11,12,13].

Taking into consideration the high prevalence of BKPyV in the population and its genetic variations, the aim of this study was to identify BKPyV infections in DBDs and examine the molecular characterization of the variants obtained. In addition, a variety of clinical and inflammatory parameters were compared, in order to determine whether any of them could be used as a marker indicating BKPyV infection.

## 2. Results

### 2.1. Genotypes

**Figure 1:**
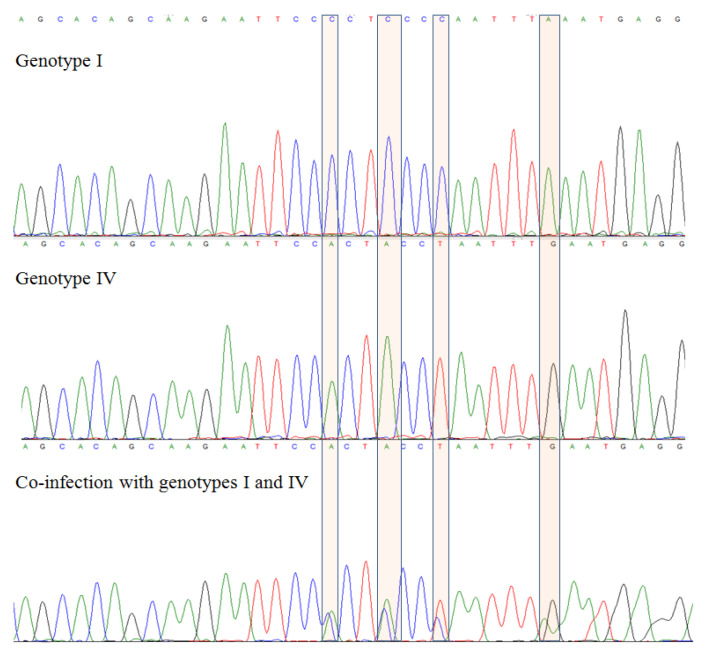
Chromatograms showing polymorphic nucleotides between genotypes I and IV and a chromatogram indicating co-infection with genotypes I and IV.

The results are in accordance with previous publications [14] and indicate a high prevalence of BKPyV in the Polish population. In our study, the genetic material of the virus was detected in 48% of donors’ urine samples, including 47% of female and 48% of male (49/103, 16/34 and 33/69, respectively—Table 1). No BKPyV genetic material was detected in any of the sera. Analysis of chromatograms and the sequences obtained revealed that the variants belonged to two genotypes, I and IV, differing in 22 nucleotide positions in the sequences (G1704A, C1716T, C1722T, G1744A, A1746T, A1747G, T1760A, A1769G, G1770A, G1775C, A1784C, A1787C, A1792G, G1793A, C1848A, C1851A, C1854T, A1860G, C1869T, G1890A, A1905G, and C1912A). Moreover, chromatogram analysis revealed co-infection with genotypes I and IV in 8.2% of DBDs (4/49) (Figure 1). These co-infections were not included in the detailed analysis at the genotype, subtype, and variant level, but their presence was taken into account in analysing the entire BKPyV-positive subgroup. Based on polymorphism analyses in the sequence encoding the VP1 protein, BKPyV with genotype I were assigned to subtype Ib-2, within which two variants were distinguished, previously described by Furmaga et al. and submitted to GenBank Database [14] as Ib-2_POL_K (G1809A) and Ib-2_POL_F (G1809C). The distribution of variants showed that Ib-2_POL_K was present in more than 62% of carriers of genotype I, while Ib-2_POL_F was present in 38% of them (13/21 and 8/21, respectively).

BKPyV infection prevalence was the highest in the age group 40–59, both in male and female (63% and 64%; and 10/16 and 21/33, respectively) and notably lower (16%; 8/49) in younger age groups (18–29 years). As referred in previous research [14], the variant designated as Ib-2_POL_K (G1809A) was more prevalent in older age groups, whereas the prevalence of Ib-2_POL_F (G1809C) was more evenly distributed among all DBD age groups. In our sample, genotype IV cases (49%; 24/49), exceeded the cases of genotype I infections (43%; 21/49). We also noted a possible age preference regarding genotype IV infections. More than 58% of the cases were in DBDs older than 50 years and it is the only genotype involved in infections over 60 years of age (Table 2). Viral load was measured within the range of 10^2^–10^7^ copies/mL (mean value 1.92 × 10^6^ copies/mL) and was notably higher in female than in male (4.68 × 10^6^ and 5.86 × 10^5^ copies/mL, respectively; *p* = NS), mainly due to the single case of a 45-year-old female with a viral titre of 7.1 × 10^7^ copies/mL. There were no statistically significant differences in the viral load either between genotypes I and IV (4.25 × 10^6^ and 2.02 × 10^5^ copies/mL, respectively; *p* = NS) or between variants Ib-2_POL_K and Ib-2_POL_F (6.33 × 10^6^ and 8.73 × 10^5^ copies/mL, respectively; *p* = NS). The highest viral load was noted in DBDs aged 30–39 and 40–49 and was an order of magnitude higher than in younger donors aged 20–29 and two orders of magnitude higher than in DBDs over 50 years old (*p* = NS).

**Figure 2:**
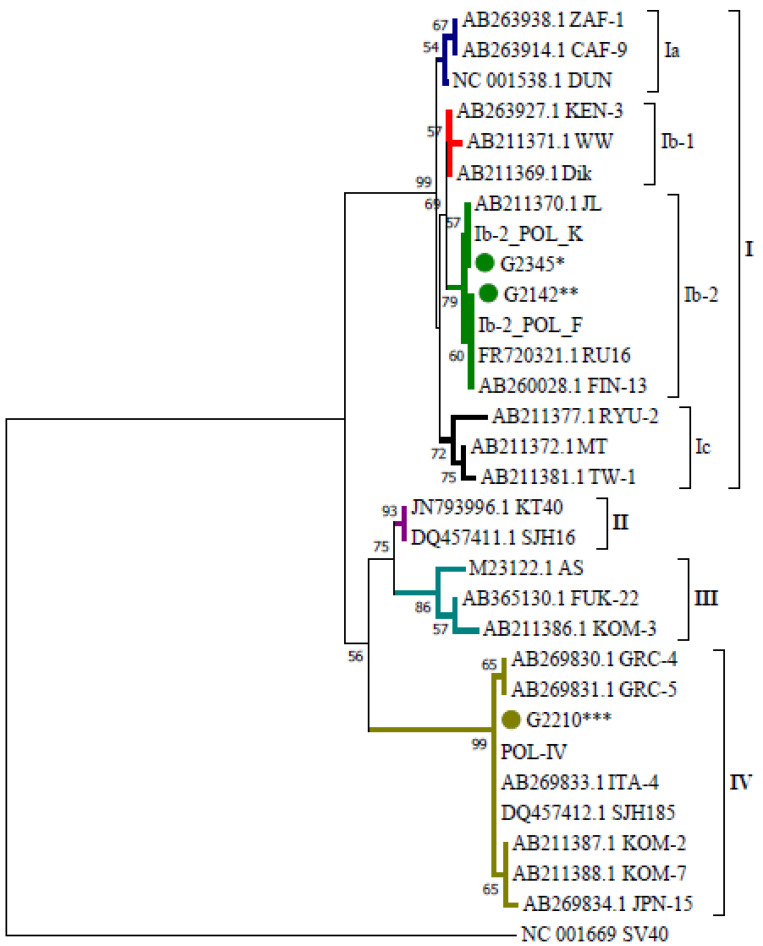
Phylogenetic tree presenting relationships between variants obtained in the study and sequences from the GenBank database. Phylogenetic analysis was carried out in MEGA11 software using the neighbour-joining (NJ) algorithm with Kimura’s two-parameter distance method and a bootstrap value of 1000. The analysis was based on a 287 bp fragment of the VP1 coding sequence. * Variant carried by G2345, G2375, G2120, G2106, G2303, G2278, G2057, G2319, G2390, G2054, G2356, G2392, G2310. ** Variant carried by G2142, G2389, G2342, G2101, G2353, G2321, G2015, G2105. *** Variant carried by G2210, G2061, G2021, G2223, G2218, G2362, G2119, G2315, G2047, G2020, G2041, G2071, G2294, G2107, G2014, G2371, G2273, G2091, G2283, G2379, G2330, G2199, G2219, G2240.

Phylogenetic analysis confirmed that BKPyV assigned to the genotype I belonged to subtype Ib-2 and that variants Ib-2_POL_K and Ib-2_POL_F belonged to two different clades within the same subtype (bootstrap value 79—Figure 2). Polymorphism G1809A, present in Ib-2_POL_K, is nonsynonymous with the DUN reference strain and involves an E82D change in the amino acid sequence, while the G1809C transversion in the case of Ib-2_POL_F is synonymous.

Within genotype IV the phylogenetic tree confirms clustering of 24 of our viral sequences with sequences from the NCBI database belonging to this genotype (including 100% similarity to POL-IV); however, the sequence of the analysed fragment does not allow this variant to be definitively classified to a subtype within genotype IV.

### 2.2. Biomarkers

Biomarkers play an important role in current diagnostics, determination of the endpoints and prediction of the clinical outcome of infection. Several studies have attempted to identify biomarkers for diseases such as dengue [15], COVID-19 [16], or H1N1 influenza [17].

Among the parameters analysed in the study, no markers specific for BKPyV infection in the DBD population were identified. Groups BKPyV (+) and BKPyV (−) did not differ significantly (*p* = NS) in clinical parameters (blood group, Rh, BMI, diurnal diuresis, length of stay in ICU, cause of death), morphological parameters (HGB, HCT, RBC), or biochemical parameters (creatinine, urea, total protein, albumin) (Supplementary materials Table S1). Additionally, the parameters of inflammation (CRP, leukocytes, lymphocytes, monocytes, NLR, dNLR, MLR) and clotting disorders (PLT; Supplementary materials Table S2) were compared between both groups, however the results did not resolve the fundamental question of whether there is a marker to conclusively identify DBDs infected with BKPyV. Therefore, despite the small size of the group of infected donors, an attempt was made to characterize individual BKPyV genotypes and variants within subtype Ib-2 (Supplementary materials Tables S3–S5). In addition, two DBDs subgroups were distinguished on the basis of differences in viral load (viral load <10^5^ and >10^5^ copy/mL; Supplementary materials Table S6), and clinical, morphological and inflammatory parameters were compared between them.

There was similar prevalence in the distribution of genotypes in DBDs and no significant difference in clinical parameters (*p* = NS). The examination of morphological and biochemical parameters revealed differences between genotypes I and IV. DBDs with genotype I were discovered to have more favourable morphological parameters (HGB, HCT, RBC; *p* = NS) and less favourable biochemical parameters (creatinine, urea; *p* = NS), while parameters of inflammation varied (elevated CRP and decreased NLR, dNLR, PLR, MLR; *p* = NS). Analysis of other tested clinical parameters also showed no statistically significant differences between them (*p* = NS).

**Figure 3:**
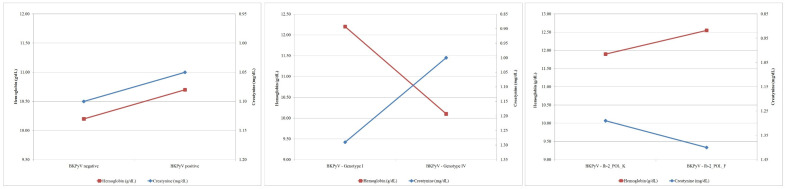
Comparison of hemoglobin and creatinine values between the BKPyV positive and BKPyV negative groups, genotypes I and IV and variants within the Ib-2 subtype.

A similar analysis was carried out for variants within subtype Ib-2 showed that DBDs with Ib-2_POL_F had more favourable morphological parameters (HGB, HCT, RBC; *p* = NS) and less favourable parameters of kidney function (elevated creatinine and urea, with lower diuresis, *p* = NS), with high values of clotting parameters (the highest PLT; *p* = NS) and inconclusive parameters of inflammation (elevated CRP, neutrophils, and lymphocytes and lower NLR, PLR, MLR; *p* = NS). Clinical parameters did not differ significantly between variants of Ib-2 (*p* = NS). Despite of the lack of the statistical significance, there is a noticeable difference in hemoglobin and creatinine values between the genotypes and variants within the Ib-2 subtype (Figure 3).

Potentially impaired kidney function was observed in the group with high viral replication in urine (≥ 10^5^ copies/mL—8/49, 16%—virus was not detected in any of the plasma samples), which was manifested as the lowest diuresis (2700 mL/day; *p* = NS) and elevation of creatinine value to 1.18 mg/dL (*p* = NS); however, without the expected negative effect on the function of the red blood cell system (anemia). Parameters of inflammation and clotting in this group showed discrepancies and no characteristic features for a marker of BKPyV infection.

Comparison of parameters of inflammation revealed significant statistical differences for CRP values between the genotypes and the uninfected DBD group (Supplementary materials Table S2). Comparison of the levels of particular cell populations of the white blood cell system and thrombocytes did not reveal differences between BKPyV-positive and BKPyV-negative groups or between the genotypes (I and IV) and the BKPyV-negative group (Supplementary materials Table S2). Similarly, the analysis of values of parameters calculated from the elements of the white blood cell system and thrombocytes, i.e., NLR, dNLR, MLR and PLR, revealed no differences either between BKPyV-positive vs. BKPyV-negative DBDs or between the genotypes and the BKPyV-negative group. A tendency towards statistical significance was observed for dNLR between medians in the BKPyV-positive vs. BKPyV-negative groups (*p* = 0.082; Supplementary materials Table S2).

## 3. Discussion

In the present study, the results of the molecular analysis confirmed the high prevalence of BKPyV genetic material (48%) in urine from DBDs, confirming that BKPyV is common in the Polish population.

There is a large amount of data confirming the high prevalence of antibodies against BKPyV, which exceeds 90% in certain populations [18,19]. The detected frequency of viruria is usually lower among healthy people and mainly affects older groups, varying from 6% to more than 40% [20,21,22], and it increases particularly in patients undergoing immunosuppression, reaching over 50% in kidney transplant recipients [23,24,25,26,27]. Nevertheless, current reports [28] indicate a significantly higher prevalence of viruria, confirming the presence of BKPyV genetic material in the urine of over 90% of donors and in nearly 90% of recipients. According to literature data and our expectations, the prevalence of BKPyV infections detected in the group of DBDs should increase with age, which in the present study was confirmed in the case of genotype I and variant Ib-2_POL_K, but not in the case of genotype IV. Identification of Ib-2_POL_F showed a fairly stable low presence in all age groups, as reported in our previous work [14].

Studies of molecular epidemiology of BKPyV indicate a varied distribution of individual genotypes and subtypes in different geographic regions. Ikegaya et al. (2007) suggested that it was possible to use genotyping of the virus in order to determine the geographic origin of an unidentified corpse [29]. The most commonly detected genotype in Europe is genotype I, whose dominance has been confirmed in countries such as England and Germany, where it exceeds 90% [30,31]. Genotype IV is most often detected in East and South Asia [32]. Nevertheless, a significant percentage of genotype IV has been noted as well in some European countries. Previous studies of the European population have shown that genotype IV was detected in 39% of subjects [33], while Momynaliev et al. [34] detected the presence of genotype IVc-2 in 24% of subjects who had received a kidney transplant. A similar, high prevalence of genotype IV has been recorded in Finland, Greece, and Hungary (45%, 50%, and 67%, respectively) [33], a finding that may indicate that despite the high frequency of genotype I in Europe, there may be areas inhabited by populations with established variants belonging to genotype IV, and a more detailed analysis of these populations might help in identification of local endemic BKPyV variants. The distribution of genotypes in our study indicates a similar frequency of genotypes I and IV in the viral pool among DBDs in Poland, while no representatives of genotype II or III were detected.

The first classification of BKPyV into genotypes was described in 1993 by Jin et al. [35] who detected the restriction sites specific for a given genotype in the variable region of the gene encoding the VP1 protein, which were detected by restriction fragment length polymorphism (RFLP). Nowadays, genotyping of the BKPyV is mostly based on the sequencing of the polymorphic fragment of BC loop (VP1 protein) which contains the region with nucleotides at positions 1744–1812 used for identification of the four main BKPyV genotypes [36,37]. Sanger sequencing is still used successfully for BKPyV genotyping due to the relatively short length of the sequence used to classify the virus into subtypes [38,39].

In this study, we confirmed a G1809A/C polymorphism in subtype Ib-2 with respect to the reference strain DUN, which made it possible to distinguish variants Ib-2_POL_K and Ib-2_POL_F, occupying separate branches within the same clade with a bootstrap value of 79. We did not detect any Ia or Ib-1 isolates, which are present in Europe (England, the Netherlands), or Ic occurring in Germany [40].

The analysis of the sequences revealed co-infection with genotypes I and IV in 8% of DBDs (4/49). Co-infections with different genotypes have previously been described by Muñoz-Gallego et al. [41], who observed mixed genotypes in more than half of patients tested (33/63), among which the most common was the combination of genotypes I and II. The researchers noted that patients with mixed genotypes developed a detectable BKPyV viral load in blood (VL > 1000 copies/mL) more rapidly and had a greater risk of clinical manifestations associated with BKPyV infection. The small number of donors with mixed genotypes in our study did not allow us to derive definitive conclusions.

In our study, the average number of copies in the majority of the urine samples (except of one case) did not exceed 1 × 10^7^ copies/mL, the value indicated by Shen et al. [42] as the urine threshold value for patients with kidney transplant. Currently, there is no established threshold for donors, so we could not relate to any known point of reference. Nevertheless, it is important to monitor the viral load, not only in recipients but also in donors, as it may be a valuable predictor of the outcome of transplantation.

The number of copies of the virus detected differed between genotypes and it was higher in genotype I than in genotype IV, but without statistical significance (4.25 × 10^6^ and 2.02 × 10^5^ copies/mL, respectively; *p* = NS). A similar relationship was observed between variants Ib-2: POL_K and POL_F, with the former being associated with more copies (6.33 × 10^6^ and 8.73 × 10^5^ copies/mL, respectively; *p* = NS).

Additionally, in our research we used simple and non-invasive methods to calculate cellular indicators of inflammation, such as NLR, dNLR, MLR and the hypercoagulable state (PLR) for all groups of DBDs [9]. These ratios are correlated with poor prognosis in a variety of conditions, including end-stage kidney disease, heart disease and cancer [10]. Previous studies have shown there is a relationship between high NLR and PLR values and the onset of acute rejection of the transplanted organ [6]. Additionally, elevated preoperative NLR values in the recipients are associated with delayed graft function following kidney transplantation [43]. Serial monitoring of these ratios was helpful in identification of subclinical inflammation, prior to other evidence of allograft dysfunction [44]. In the recipient, the high preoperative NLR value can also influence the function of the graft immediately after the operation [43]. However, what is worth noting, is the tendency observed in the present study, that indicates higher dNLR values in BKPyV-negative donors compared to BKPyV-positive donors (*p* = 0.082). The lack of statistical significance is consistent with observations suggesting a low prognostic value of parameters derived from blood in predicting inflammation following a kidney transplant [45]. NLR and dNLR may therefore have only supplementary diagnostic value in relation to a basic white blood cells count. It seems that only the combination of NLR and the levels of total protein and urea in the serum can be a accurate negative predictor following the kidney transplantation [45].

The small number of donors examined in our study undoubtedly constitutes a serious limitation and we do not confirm the hypothesis that the aforementioned ratios could be used alone as reliable markers identifying BKPyV infection in DBDs. Furthermore, there is insufficient literature data to answer this clinically important question. More studies need to be conducted in order to determine whether a high BKPyV titer in donors affects the function of the transplanted organ and the outcome of the graft and recipients, and what is the threshold value of BKPyV titer in donors, which genotypes and variants of BKPyV are the most threatening to the graft, and what simple markers of systemic cellular inflammation can be useful in stratifying the risk of transplanting a BKPyV-infected organ.

## 4. Materials and Methods

### 4.1. Study Population

From January 2013 to March 2018, in cooperation with the First Chair and Department of General and Transplant Surgery and Nutritional Treatment, Medical University of Lublin (Poland), in accordance with the provisions of Polish law, 149 cases of brain death in patients were diagnosed, and all of the deceased were reported as potential donors to the central database in Warsaw at the Polish Transplant Coordinating Centre (Poltransplant). All of the 149 DBDs were pre-qualified for the study. In the absence of objections to organ donation in the Central Register of Objections, submitted by the deceased while alive and/or by law enforcement agencies, and following the family’s consent to further medical procedures in compliance with Polish law, the deceased were examined for the presence of severe infections (bacterial, viral, fungal or others), cancer and significant metabolic or hematological disease. On this basis, 46 of the deceased were disqualified from being donors. Organs from the remaining 103 DBDs were ultimately transplanted to recipients. Multiple organs were used in 57% of cases (59/103), and only kidneys were taken from the remaining 43% (44/103). In total, 299 organs (including 206 kidneys) were collected, of which 298 were transplanted. Finally, 166 kidneys were transplanted at the First Chair and Department of General and Transplant Surgery and Nutritional Treatment in Lublin, Poland. Two out of 3 DBDs were male (69/103) with an average age of 43 years (Table 3). The cause of death was craniocerebral trauma, vascular events (hemorrhagic or ischemic stroke), and secondary brain injuries such as brain oedema, sudden cardiac arrest or poisoning (47%, 42%, and 11%, respectively).

Urine and plasma samples were collected from each DBDs directly before harvesting the organs, in addition to the standard laboratory tests performed in such cases. After initial preparation, the samples were stored at −80°C until further analysis was performed. Ethical approval for this research (KE-0254/281/2017) was granted by the Bioethics Committee of the Medical University of Lublin, Poland.

### 4.2. DNA Extraction and PCR Amplification

DNA was extracted from the blood and urine with the DNeasy Blood & Tissue Kit (Qiagen, Hilden, Germany) as previously described [14]. After extraction, each DNA sample was verified by amplification with primers specific to human β-actin [46] to avoid false negative results, which can result from ineffective amplification or the presence of PCR reaction inhibitors. Amplification was carried out with primers PC03_F (5′ ACACAACTGTGTTCACTAGC 3′) and PC04_R (5′ CAACTTCATCCACGTTCACC 3′). The reaction mixture contained 1 U Taq polymerase AmpliTaq Gold 360 DNA Polymerase (Applied Biosystems, Foster City, CA, USA) in the manufacturer’s buffer, adjusted to a final concentration of 2.5 mM MgCl_2_, 0.8 mM of each dNTP, and 0.8 mM of each primer—25 μL total volume. The reaction took place under the following conditions: 95 °C for 10 min, 36 cycles of 95 °C for 45 s, 56 °C for 45 s, 72 °C for 45 s, and 72 °C for 10 min in a Labcycler thermocycler.

PCR was used to detect BKPyV genetic material in the samples. The primers used amplified a partial sequence of the VP1 protein, spanning from 1630 bp to 1956 bp in sequence NC_001538 from the NCBI database [47]. The reaction was carried out with primers 327-1PST (5′ CAAGTGCCAAAACTACTAAT 3′) and 327-2HIN (5′ GCATGAAGGTTAAGCATGC 3′) targeting the typing region used for genotyping of BKPyV variants. The composition of the reaction mixture for these primers was the same as for amplification of β-actin. However, the reaction conditions were slightly modified: 95 °C for 10 min, 40 cycles of 95 °C for 45 s, 54 °C for 45 s, 72 °C for 45 s, and 72 °C for 10 min.

PCR products were separated in a 2% agarose gel with ethidium bromide at 70 V. The lengths of the bands were determined by comparison to GeneRuler 100 bp size markers (Thermo Fisher Scientific, Foster City, CA, USA). The electrophoresis results were analysed under UV light with Scion Image software (Scion Corporation, Frederick, MD, USA).

### 4.3. Sequencing and Bioinformatic Processing

The sequencing reaction was carried out in a 3100 Avant Genetic Analyser (Applied Biosystems) using the BigDye^®^ Terminator 3.1 CycleSequencing Kit (Thermo Fisher Scientific, Foster City, CA, USA) as previously described [14]. Sequencing results were analysed using DNA Baser software. Editing, alignment of sequences, localization of polymorphisms and assignment to types were performed using MEGA 11 and Bioedit software. Phylogenetic analysis was carried out using MEGA11 by the neighbor-joining (NJ) algorithm with Kimura’s two-parameter distance method and a bootstrap value of 1000.

BKPyV genotypes were determined by comparing the sequences obtained to sequences from the NCBI database. BKPyV variants were classified into genotypes on the basis of polymorphic nucleotides proposed by Randhawa et al. [47], using an algorithm designed by Morel et al. [39] and phylogenetic analysis based on nucleotide sequences.

### 4.4. Evaluation of Viral Load

Qualitative assessment of BKPyV was conducted by qPCR, using the GeneProof BK/JC Virus PCR Kit in the ABI Prism^®^ 7500 Real-Time PCR System (Applied Biosystems), according to the kit manufacturer’s instructions. BKPyV detection is based on amplification of a specific conservative DNA sequence overlapping the boundary between the genes for the VP1 and VP2 proteins. The presence of viral genetic material is indicated by the increase in FAM fluorophore fluorescence. The reaction was prepared in a 40 μL volume (30 μL of Reaction Mix and 10 μL of DNA). The qPCR reaction conditions were as follows: UDG incubation at 37 °C for 2 min, DNA hot start polymerase activation at 95 °C for 10 min, followed by 45 cycles of DNA melting at 95 °C for 5 s, annealing at 60 °C for 40 s, and extension at 72 °C for 20 s.

Serial dilutions of 2×10^4^ copies/μL, 2 × 10^3^ copies/μL, 2 × 10^2^ copies/μL, and 2 × 10^1^ copies/μL were used to prepare the standard curve, which was used to determine the number of copies of the virus based on the Ct value. Negative control reactions without DNA and a positive control supplied by the manufacturer were also included in every run. The samples were run in duplicate. Viral load was expressed as the number of BKPyV copies per mL.

### 4.5. Blood Cell Count and Derived Ratios

Different leukocyte subtypes and platelet counts were obtained using an automated ADVIA 2120 hematology analyzer (Siemens Healthcare Diagnostics, Deerfield, IL, USA).

The NLR was calculated as quotient of the absolute number of neutrophils to the absolute number of lymphocytes. The dNLR value is calculated using the formula: dNLR = neutrophils/(leukocytes-neutrophils) [48]. MLR was calculated as quotient of the absolute number of monocytes to the absolute number of lymphocytes. Additionally, PLR was calculated as quotient of the absolute number of thrombocytes to the absolute number of lymphocytes [44].

### 4.6. Statistical Analysis

All data were analysed using Statistica 13 software (TIBCO Software Inc., Palo Alto, CA, USA).The hypothesis of normal distribution was verified for each data set using the Shapiro–Wilk test. The equality of variances was verified using Levene’s test. Due to the lack of normal distribution Mann–Whitney U test was performed to compare the parameters between BKPyV positive and negative groups. A nonparametric Kruskal–Wallis test was reported for comparisons between particular genotypes or subtypes and BKPyV negative groups. Statistical significance was set at *p* ≤ 0.05.

## 5. Conclusions

The results confirm high incidence of BKPyV in the study population, a relatively high proportion of samples classified as genotype IV, and the possibility of simultaneous co-infection with two genotypes of the virus. Molecular analysis of the BKPyV indicates the existence of two main variants within subtype Ib-2, distinguished on the basis of a polymorphism (G1809A/C): Ib-2_POL_K and Ib-2_POL_F.

BKPyV was detected more often in older age groups, and reached the highest viral load in the 30–39 and 40–49 age groups. Nowadays, none of the clinical parameters and cellular indicators of inflammation is being used as a specific marker for BKPyV infection. The recommended parameter used in order to make inferences about the course of BKPyV infection and to determine the treatment strategy has been the number of copies of the virus. Our results, however, indicate that the genotype of the virus is also a valuable predictive marker of the course of infection. Among the genotypes tested, infection with genotype I showed a tendency to adversely affect kidney function (*p* = NS), while infection by genotype IV was mainly associated with variable degree of anemia (*p* = NS). This is of great importance, as it can affect not only recipients of kidneys from DBDs, but also recipients of hematopoietic stem cell transplantation (HSCT) and kidneys from living donors. In view of the above, the level of viruria itself may be insufficient to make inferences on the pathogenicity of BKPyV.

There is an unquestionable need for further research on genetic variation in BKPyV and its effect on the clinical course of infection. A better understanding of the molecular background of infection and the virus’s mechanisms of adaptation may contribute to better inferences about the course of infection and better therapeutic management.

## Figures and Tables

**Table 1:** Distribution of genotypes and subtypes broken down by sex.

	BKPyV Positive				Total BKPyV Negative	Total
	Genotype I (Ib-2_POL_K/Ib-2_POL_F)	Genotype IV	Genotype I and IV (Co-Infection)	Total BKPyV Positive		
M	16 (10/6)—(23.19%–14.49%/8.70%)	14 (20.29%)	3 (4.35%)	33 (47.83%)	36 (52.17%)	69 (100%)
F	5 (3/2)—(14.71%–8.82%/5.88%)	10 (29.41%)	1 (2.94%)	16 (47.06%)	18 (52.94%)	34 (100%)
Total	21 (13/8)—(20.39%–12.62%/7.77%)	24 (23.30%)	4 (3.88%)	49 (47.57%)	54 (52.43%)	103 (100%)

**Table 2:** Distribution of genotypes, subtypes and viral loads broken down by age and sex.

			Average Viral Load (Copies/mL)			FREQUENCY			
Sex	Age	Number	Average Number of Copies	Genotype I	Genotype IV	Subtype-Variant			
						Ib-2_POL_K	Ib-2_POL_F	POL-IV	Co-Infection
M	<20	2 (6.06%)	3.07 × 10^3^	-	8.30 × 10^2^	-	-	1	1
	20–29	3 (9.09%)	3.37 × 10^3^	4.86 × 10^3^	4.10 × 10^2^	1	1	1	-
	30–39	5 (15.15%)	1.52 × 10^6^	2.32 × 10^6^	3.25 × 10^5^	-	3	2	-
	40–49	9 (27.27%)	1.27 × 10^6^	2.25 × 10^6^	6.46 × 10^4^	4	1	3	1
	50–59	12 (36.36)	2.01 × 10^4^	7.75 × 10^3^	3.88 × 10^4^	5	1	5	1
	60–69	2 (6.06%)	2.45 × 10^3^	-	2.45 × 10^3^	-	-	2	-
Total		33	5.86 × 10^5^	1.14 × 10^6^	7.46 × 10^4^	10 (30.30%)	6 (18.18%)	14 (42.42%)	3 (9.09%)
F	<20	-	-	-	-				
	20–29	3 (18.75%)	1.24 × 10^6^	-	1.24 × 10^6^	-	-	3	-
	30–49	-	-	-	-				
	40–49	3 (18.75%)	2.37 × 10^7^	2.37 × 10^7^	-	2	1	-	-
	50–59	7 (43.75%)	1.27 × 10^4^	2.12 × 10^3^	7.11 × 10^3^	1	1	4	1
	60–69	3 (18.75%)	1.77 × 10^4^	-	1.77 × 10^4^	-	-	3	-
Total		16	4.68 × 10^6^	1.42 × 10^7^	3.81 × 10^5^	3 (18.75%)	2 (12.50%)	10 (62.50%)	1 (6.25%)
M and F	<20	2 (4.08%)	3.07 × 10^3^	-	8.30 × 10^2^	-	-	1	1
	20–29	6 (12.24%)	6.23 × 10^5^	4.86 × 10^3^	9.33 × 10^5^	1	1	4	-
	30–39	5 (10.20%)	1.52 × 10^6^	2.32 × 10^6^	3.25 × 10^5^	-	3	2	-
	40–49	12 (24.49%)	6.87 × 10^6^	1.03 × 10^7^	6.46 × 10^4^	6	2	3	1
	50–59	19 (38.78%)	1.74 × 10^4^	6.34 × 10^3^	2.47 × 10^4^	6	2	9	2
	60–69	5 (10.20%)	1.16 × 10^4^	-	1.16 × 10^4^	-	-	5	-
Total		49	1.92 × 10^6^	4.25 × 10^6^	2.02 × 10^5^	13 (26.53%)	8 (16.33%)	24 (48.98%)	4 (8.16%)

**Table 3:** Characterization of test group by age, sex and BMI.

		BKPyV (+)		BKPyV (−)		Total		*p*-Value
Factor	Sex	Positive, *n* = 49		Negative, *n* = 54		*n* = 103		
		Mean	STD	Mean	STD	Mean	STD	
Age	M	43.55	13.34	37.53	14.25	40.41	14.05	0.708
	F	48.88	15.35	44.67	14.15	46.65	14.66	0.741
	Total	45.29	14.09	39.91	14.49	42.47	14.48	0.849
BMl	M	25.57	3.80	25.51	4.04	25.54	3.90	0.734
	F	24.99	5.26	25.84	5.47	25.44	5.31	0.883
	Total	25.38	4.28	25.62	4.52	25.50	4.39	0.712

## Data Availability

We have included all additional data as Supplementary materials.

## Supplementary Materials

The following supporting information can be downloaded at: https://www.mdpi.com/article/10.3390/ijms23169173/s1.
